# Enhanced adaptive control techniques for extracting maximum power from photovoltaic system

**DOI:** 10.1038/s41598-025-26330-4

**Published:** 2025-11-23

**Authors:** Ahmed O. Hafez, Mahmoud A. Attia, Ahmed H. EL-Ebiary

**Affiliations:** Department of Electrical Power and Machines, Faculty of Engineering, Ain Shams University, Cairo, Egypt

**Keywords:** Energy science and technology, Engineering

## Abstract

Photovoltaic (PV) systems experience performance fluctuations due to changes in irradiance and temperature, which makes maximum power point tracking (MPPT) essential for stable grid integration. This paper investigates four adaptive MPPT controllers integrated with the incremental conductance (IC) algorithm: (i) a PID controller optimized by manta ray foraging optimization (MRFO), (ii) an Adaptive PI (API) controller, (iii) a Single Perceptron Adaptive PI (SP-API) controller, and (iv) a Set Membership Affine Projection Algorithm (SMAPA)-based PI controller. Unlike conventional or offline-trained approaches, the proposed controllers adapt in real time to environmental changes without requiring large datasets. The main novelty lies in applying SP-API and SMAPA for MPPT in PV systems for the first time, and in enhancing PID and API controllers with MRFO-based tuning for improved robustness. To evaluate performance, two case studies are conducted on a grid-connected PV system: 1- uniform irradiance and temperature variations to simulate daily operating conditions, and 2- partial shading scenarios to test adaptability under local irradiance mismatches. Results show that the SMAPA-based PI controller achieves near-ideal efficiency (~ 99.8%), minimal ripples, and negligible energy losses (~ 0.2%), followed by the SP-API controller. In contrast, the conventional PID and API controllers demonstrate weaker dynamic responses and higher losses. These findings confirm that SMAPA and SP-API offer superior adaptability and stability, making them promising solutions for reliable MPPT in grid-connected PV systems.

## Introduction

Photovoltaic (PV) energy, in general, is considered as an alternative source of energy due to its abundance, and for being eco-friendly. The everyday growing economy and its dependency on fossil-based energy production has had a dire effect on the environment and resulting in an alarming level of greenhouse gas emission. According to the latest global and CO2 status report by the International Energy Agency (IEA), the total energy demand in the world has grown by 2.3% and electricity generation has grown by 4%^1^. But Renewables, in general, have seen an increasing interest throughout the world. Total energy demand is the total energy that is required throughout the world and electricity generation is the total energy produced by all energy sectors. PV systems are being used in many real-world applications like irrigation, home power supply, and commercial electric car charging stations^2^. PV systems tied to the grid face a lot of challenges related to weather and environmental changes and non-linearities in PV systems components which directly affect the performance of the PV system, the extracted energy and maximum power that can be delivered to the grid requires online adaptive control systems to continuously adapt to the different weather conditions. The integration of a PV system with the utility grid is achieved using a DC–DC boost converter and a DC–AC inverter. The boost converter is regulated by MPPT techniques to adapt to environmental variations.

The two commonly used methods are Perturb and Observation (P&O) and Incremental Conductance (IC)^3–5^. Many researchers have worked on improving IC performance by modifying the controllers used in grid-tied PV systems. Although the IC algorithm can quickly drive the operating point toward the maximum power point (MPP) during sudden disturbances, it suffers from poor dynamic performance and introduces sustained oscillations around the MPP. The IC is enhanced by introducing a variable adjustment mechanism based on an integral regulator. This enhanced method is Integral Regulator & Incremental Conductance (I&IC) algorithm^6^. Others used optimized PID controllers, cascaded controllers, fuzzy controllers and adaptive controllers to enhance the performance of the IC algorithm. Authors of^7^ proposed Fuzzy-PID MPPT, this controller employs fuzzy inference to adapt PID gains online based on error and error rate, improving tracking speed and reducing ripple compared to conventional PID and IC. However, its performance remains constrained by the inherent limitations of PID control structures. In^8^ a Neuro-Fuzzy IC MPPT is proposed, this controller integrates fuzzy logic with a neural network trained on incremental conductance data to achieve variable step-size adaptation. It delivers fast convergence with minimal oscillations, however its reliance on offline training limits robustness under unmodeled conditions. Authors of^9^ proposed a Support Vector Machine (SVM)-based MPPT controller for PV systems, using solar radiation and temperature as inputs to predict the optimal operating voltage but lacks the adaptive real-time gain tuning. In^10^ a Cuttlefish Algorithm (CFA) is proposed to enhance PV system performance under partial shading by tuning control gains through meta-heuristic optimization, it depends on offline stochastic optimization rather than ensuring bounded-error stability and online adaptability. Authors of^11^ proposed developed a Bond Graph–based MPPT controller, validated in simulation and hardware, which offers an energy-based modeling framework but depend on fixed model equations without online adaptation. Authors of^12^ data-driven artificial neural network (ANN)-based MPPT technique. Two techniques tested ANN-GT: inputs are solar irradiance (G) and temperature (T) and ANN-TI: inputs are PV current (I) and temperature (T). The ANN is trained using 6100 data samples from simulations and experiments and implemented on a microcontroller. The ANN Uses a trained feedforward neural network to learn nonlinear relations between irradiance, temperature, current, and the duty cycle at MPP and needs large dataset for offline training. In^13^ proposes a dynamic MPPT controller for photovoltaic systems that integrates Long Short-Term Memory (LSTM) neural networks with Artificial Neural Networks (ANN) and Fuzzy Logic Control (FLC) to enhance power extraction under rapidly changing irradiance and temperature conditions. By leveraging LSTM’s ability to capture temporal dependencies and ANN/FLC’s adaptive decision-making, the controller predicts future maximum power points and dynamically adjusts the DC–DC converter’s duty cycle to track them efficiently, even under partial shading. The LSTM models are trained with historical irradiance and temperature datasets (from NASA POWER project, Mar 2023–Mar 2024) to learn the relationship between environmental inputs and the voltage/current at the maximum power point Once trained, the controllers use these pre-learned models online to make predictions and guide the MPPT process, but they do not update weights or adapt continuously during real-time operation. In^14^ Model Reference Adaptive Control (MRAC) with a fuzzy logic controller is proposed to improve the performance of photovoltaic (PV) systems under varying irradiance and temperature conditions. The MRAC structure provides online parameter adjustment by continuously comparing the PV system output with a reference model, while the fuzzy controller enhances decision-making by handling system nonlinearities and uncertainties. In^15^ proposes a hybrid MPPT strategy that integrates Current Tracking Perturb & Observe (CT-P&O) with Finite Control Set Model Predictive Control (FCS-MPC) for PV-fed boost converters. The CT-P&O generates a reference current, while FCS-MPC optimizes switching states using an enhanced cost function designed to minimize inductor current ripple and improve transient performance. Authors of^16–18^ analyzed adaptive hill-climbing MPPT algorithms, emphasizing the trade-offs between conventional and modified hill-climbing techniques (P&O, INC, INR, and variable step-size versions) under varying irradiance and temperature. Their results show that adaptive and variable step-size approaches generally improve convergence speed and reduce oscillations compared to classical methods, but they may suffer from instability or fail to track the true MPP at very low irradiance, while conventional methods are more reliable yet less efficient. However, these studies did not include case studies involving multiple PV arrays or partial shading scenarios, which are critical for evaluating controller performance under conditions with multiple local peaks and the need to reliably extract the global maximum. Authors of^19^ propose a Tunicate Swarm Algorithm–Particle Swarm Optimization (TSA-PSO) method for maximum power point tracking (MPPT) in photovoltaic systems under partial shading conditions. By combining the strong exploration ability of TSA with the fast convergence of PSO, the algorithm effectively avoids local maxima and rapidly converges to the global maximum power point (GMPP). Simulation and experimental results demonstrate that TSA-PSO outperforms classical methods such as P&O, TSA, PSO, Flower Pollination Algorithm (FPA), and Grey Wolf Optimizer (GWO). Authors of^20,21^ proposed Incremental Conductance (INC) method and variable step perturb and observe (VS-PO) with a Model Reference Adaptive Controller (MRAC) based MPPT framework for photovoltaic systems, combining the Incremental Conductance method for reference voltage generation with a Model Reference Adaptive Controller to regulate the converter duty cycle. The approach ensures fast convergence, negligible oscillations, and robustness under fluctuating irradiance, temperature, and load uncertainties. Authors of^22^ introduced a hybrid two-stage adaptive MPPT scheme for PV systems to address the nonlinear challenges of tracking under variable environmental conditions. The first stage generates reference voltage, while the second stage employs a Modified Model Reference Adaptive Controller (MMRAC) to regulate the duty cycle and maintain stable operation at the MPP. Validated through stand-alone, partial shading, grid integration, and OPAL-RT experiments. Authors of^23^ present a continuous-time Lyapunov-based Model Reference Adaptive Control (LB-MRAC) method for MPPT in PV systems, where P&O generates the reference voltage and MRAC regulates the duty cycle of the boost converter. The technique is tested under stand-alone, partial shading, and grid-integrated conditions using simulations and OPAL-RT experiments. Authors of^24^ introduced a Lyapunov-based Robust Model Reference Adaptive Controller (LRMRAC) for MPPT in photovoltaic systems, designed to ensure rapid, accurate, and ripple-free tracking. By combining a P&O-based reference generator with Lyapunov-stable adaptive control, the system achieves critically damp dynamics and robustness against uncertainties. Authors of^25^ present a dual-tracking MPPT technique that perturbs both voltage and current to improve maximum power extraction from photovoltaic systems under rapidly changing irradiance and temperature. It demonstrates faster tracking, reduced oscillations, and higher efficiency compared to conventional and modified hill-climbing algorithms, especially at low irradiance levels. However, the study does not consider partial shading conditions involving multiple PV arrays, where local peaks appear in the P–V curve, making MPPT more complex. These partial shading scenarios are addressed in our study to ensure reliable tracking of the global maximum power point (GMPP) under non-uniform conditions. Authors of^26^ The paper presents a voltage and current reference-based MPPT technique that ensures fast and accurate tracking under sudden changes in irradiance and load resistance using dual perturbation, adaptive step-size reduction, and a deviation avoidance loop. It achieves high efficiency and minimal oscillations, outperforming other drift-free algorithms. However, it does not address partial shading across multiple PV arrays, where local peaks appear in the P–V curve—a condition considered in my work to ensure reliable global MPP tracking. Authors of^27^ proposes an Enhanced Model Reference Adaptive Control (EMRAC)-based MPPT method for a 100.7 kW grid-connected PV system, achieving an average efficiency of 98.28% with a response time of 0.11 s. It delivers stable power output and minimal fluctuation (~ 1 kW) under varying irradiance and temperature conditions. Experimental validation using a dSPACE 1202 platform confirms its superior tracking precision, stability, and grid compatibility. Authors of^28^ propose a Model Reference Adaptive Control (MRAC)-based MPPT technique to improve PV system efficiency under rapidly changing irradiance and temperature. The proposed controller achieves up to 99.75% tracking efficiency and reaches the MPP within 4 ms, outperforming conventional and swarm-based MPPT methods in simulations and real-time OPAL-RT tests.

From the reviewed literature, MPPT techniques can be categorized as conventional controllers, Metaheuristic Optimization (MO) controllers, Artificial Intelligence (AI) controllers, and Hybrid MPPT controllers^29–34^. Conventional Controllers are based on mathematical models. They are simple to implement but suffer from high oscillation, low efficiency and no adaptive parameters during different conditions. Metaheuristic Optimization controllers are widely used in MPPT applications. But suffer from complexity and is not preferable for real-time or online adaptations. AI and Hybrid MPPT used offline-trained controllers. These approaches require large and diverse data sets to capture the nonlinear characteristics of PV systems under varying irradiance and temperature. However, since the controller parameters are fixed after training, these methods lack online adaptation. This limits their robustness when deployed in real PV systems that are subject to rapid weather fluctuations and irradiance variance. This paper proposes online adaptive controllers. The proposed methods do not require extensive datasets for training. Instead, they continuously adapt in real time to environmental changes, ensuring fast tracking and robustness against fluctuations. The proposed adaptive controllers in this paper are integrated with the Incremental Conductance (IC) technique to enhance system performance under disturbances. The main contributions of this study can be summarized as follows:

- The integration of Incremental Conductance (IC) with four advanced adaptive controllers (PID-MRFO, API, SP-API, and SMAPA) for MPPT in grid-connected PV systems.
- The first application Set Membership Affine Projection Algorithm (SMAPA) in PV-MPPT, offering online adaptability without requiring large offline datasets.
- Enhanced robustness against dynamic irradiance, temperature variations, and partial shading, ensuring stable operation in realistic conditions.
- Comparative evaluation of multiple adaptive controllers under uniform, fluctuating, and partial shading scenarios, providing a comprehensive performance benchmark.

The five sections of the paper are as follows: Sect. 2 presents the system model; Sect. 3 presents four different controllers used in the MPPT; Sect. 4 presents two case studies applied to the system to compare the performance of the four controllers. Lastly, Sect. 5 presents the conclusion of the paper.

## PV system model

**Fig. 1:**
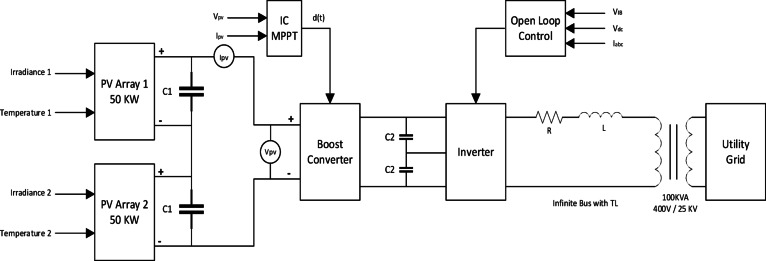
PV System tied to The Grid Block Diagram.

**Fig. 2:**
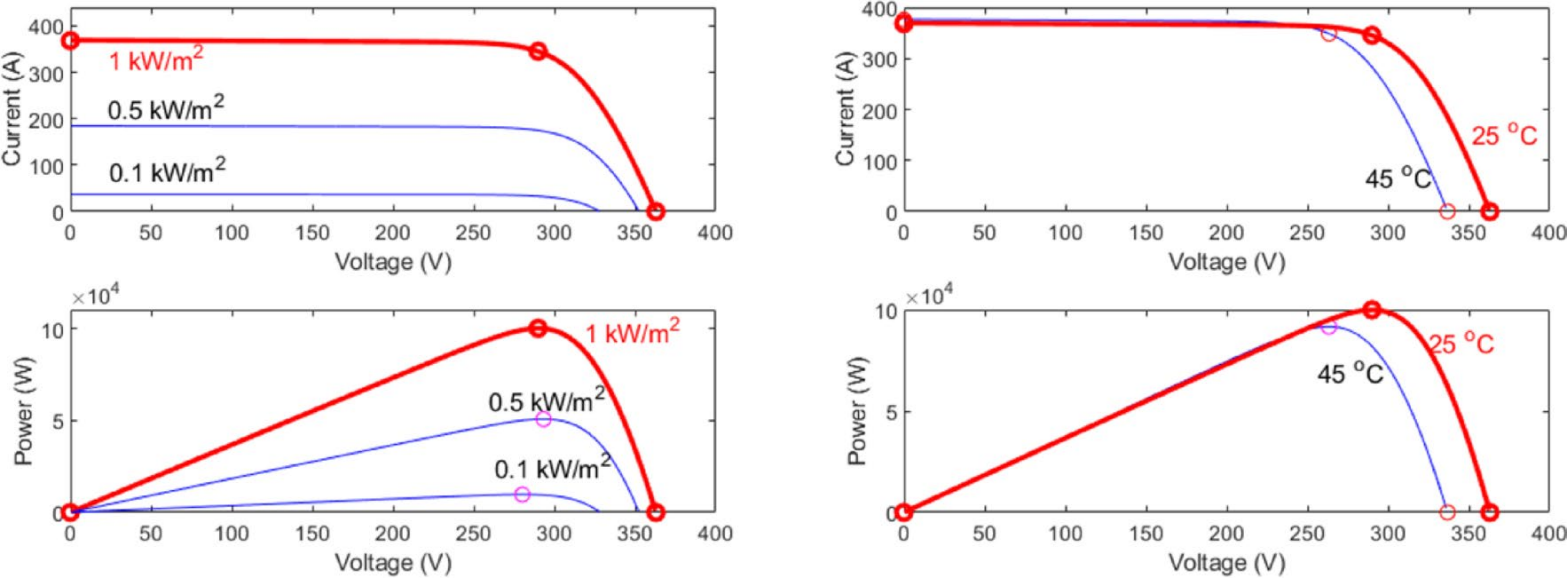
I-V and P-V characteristics for The Two PV Arrays.

The PV system is a grid tied system consisting of Two PV arrays, boost converter, inverter and step up transformer 400 V /25KV to tie to utility grid. Figure 1 Each PV array system used in this model consists of 47 parallel strings and 5 Series-connected modules per string, Module maximum power is 213.15 W, Module voltage at maximum power point $\:{\text{V}}_{\text{m}\text{p}}$ is 29 V and Module current at maximum power point $\:{\text{I}}_{\text{m}\text{p}}$ is 7.35 A Fig. 2.

**Fig. 3:**
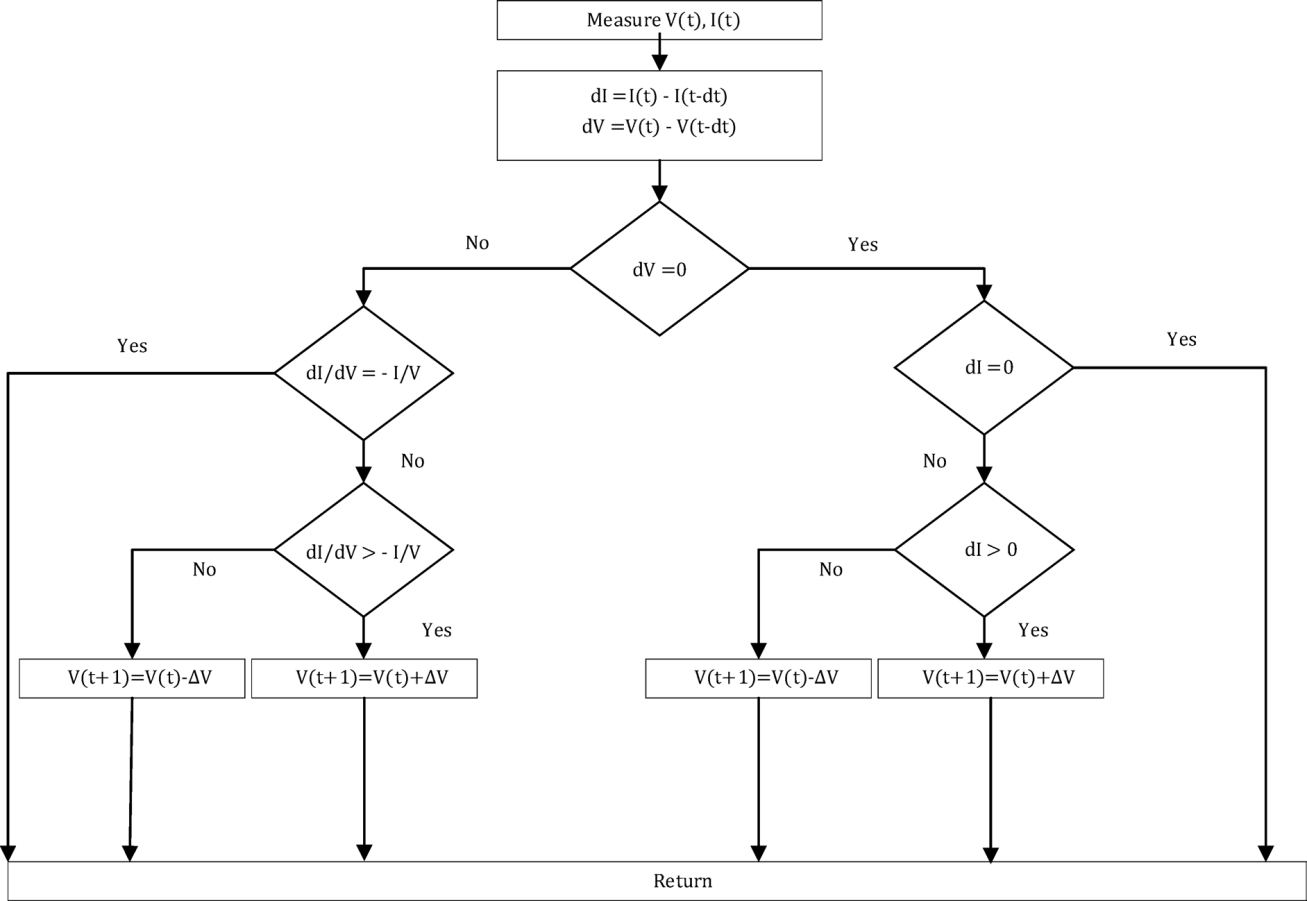
Incremental Conductance Technique.

The Incremental Conductance Technique (ICT) is one of the most widely used MPPT methods due to its simplicity and effectiveness in extracting the maximum power point (MPP). In this method, the controller continuously evaluates the incremental conductance $\:\left(\frac{dI}{dV}\right)$ and compares it with the instantaneous conductance $\:(-\frac{I}{V})$. Based on this relationship, the operating point of the PV system is adjusted to track the MPP as per (1), (2) and (3) under varying irradiance and temperature conditions as shown in Fig. 3^25^.

1 $$\:\frac{dI}{dV}\:=\:-\:\frac{I}{V}\:\text{i}\text{f}\:\:\:\:\:\text{V}\:={\text{V}}_{\text{M}\text{P}\text{P}}$$

2 $$\:\frac{dI}{dV}\:>\:-\:\frac{I}{V}\:\text{i}\text{f}\:\text{V}\:<{\:\text{V}}_{\text{M}\text{P}\text{P}}$$

3 $$\:\frac{dI}{dV}<\:-\:\frac{I}{V}\:\text{i}\text{f}\:\text{V}\:>\:{\:\text{V}}_{\text{M}\text{P}\text{P}}$$

**Fig. 4:**
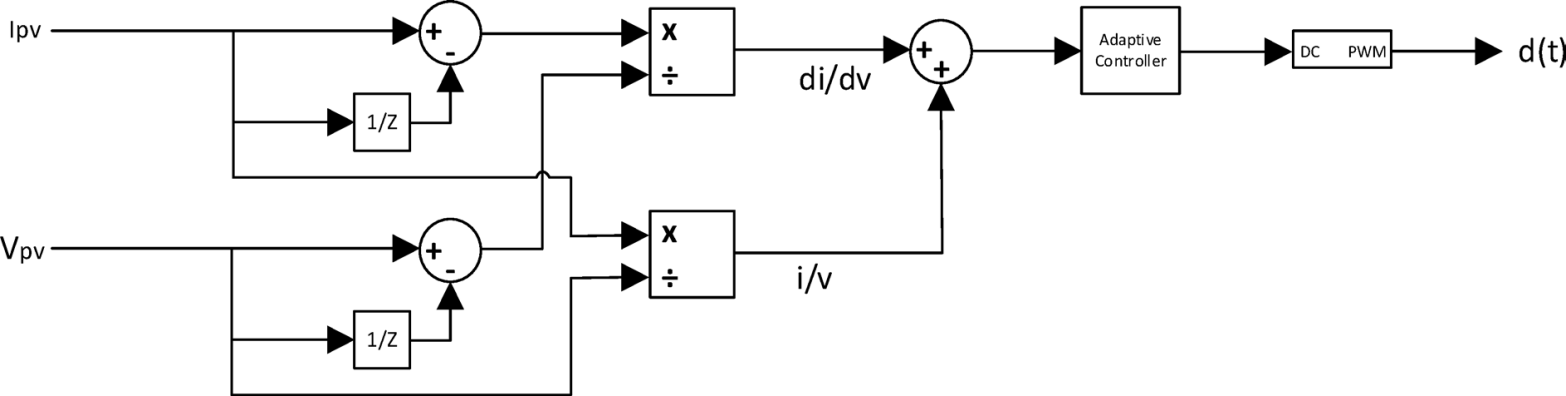
Integral Regulator Incremental Conductance controlled by Adaptive Controller.

However, it suffers from poor dynamic performance. This poor dynamic performance is highly attributed to the use of fixed steps of the duty cycle for the DC-DC converter for tracking the MPP. If the fixed step in the duty cycle is large, the MPPT will accelerate the operating point toward the MPP; however, there will be sustained oscillations around the MPP as the need precise value of the duty cycle for operation at the MPP is not found. For enhancing the ICT algorithm which will be called the Integral Regulator Incremental Conductance (I&IC) technique Fig. 4. As an enhancement to the incremental conductance technique^6^, an integral regulator was added to it to minimize the error and optimize the duty cycle correction factor. In incremental conductance technique the error equals to

4 $$\:\:\text{e}\left(\text{t}\right)\:=\:\frac{dI}{dV}\:+\:\frac{I}{V}$$

## The controllers used in MPPT

In This research to achieve a high performance from I&IC technique Different adaptive controllers used such as PID controller, adaptive PI controller, single perceptron adaptive PI controller and SPWM.

### PID controller

**Fig. 5:**
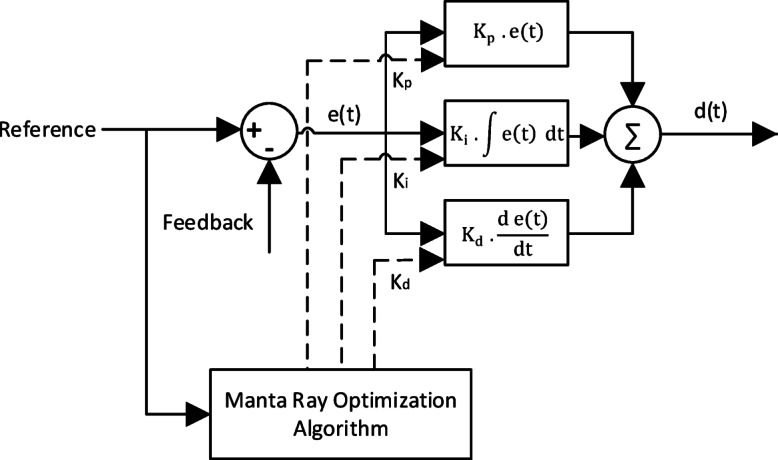
PID Controller Block Diagram.

PID Controller, considered as conventional controller, is optimized and tuned for specific duty. The gains of PID controller $\:{\text{K}}_{\text{P}}$, $\:{\text{K}}_{i}$ and $\:{\text{K}}_{d}$ are optimized by manta ray foraging optimization algorism^35^ (MRFO) Fig. 5 and the objective function for my controller is

5 $$Obj. Fn. ={\int\:}_{0}^{T}{\left(error\right(t\left)\right)}^{2}\:\:dt$$

**Fig. 6:**
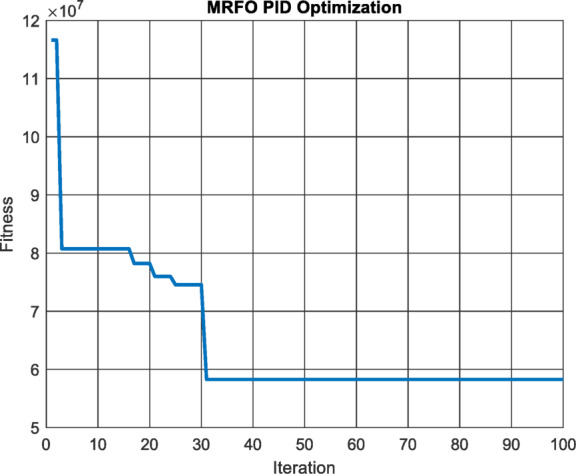
Performance vs. Iterations for PID Controller.

MRFO algorithm is used with population size 20 in 100 iterations to minimize the integral square error of the PID controller of the MPPT Fig. 6 and the results were $\:{\text{K}}_{\text{P}}=\:$2.1956, $\:{\text{K}}_{i}=\:$0.7183 and $\:{\text{K}}_{d}=\:$0.0012.

### Adaptive PI controller

Cause of continues variation of nature condition of irradiance and temperature which need online adaptation to get maximum power and best performance at any condition adaptive PI controller (API)^36,37^ may use which is give online adaptation for $\:{\text{K}}_{\text{P}}\:\text{a}\text{n}\text{d}\:$ $\:{\text{K}}_{i}\:\:$[38] So, the output of the controller is given by

6 $$\:Output\left(t\right)=\:{C}_{3}({K}_{p}\left(t\right)e\left(t\right)+\underset{0}{\overset{t}{\int\:}}{K}_{i}\left(t\right)e\left(t\right)dt)$$

and $\:{\text{K}}_{\text{P}}$ and $\:{\text{K}}_{i}$ are continuously updated by

7 $$\:{K}_{p}\left(t\right)={e}^{2}\left(t\right)+{C}_{1}\underset{0}{\overset{t}{\int\:}}{e}^{2}\left(t\right)dt$$

8 $$\:{K}_{i}\left(t\right)={C}_{2}\underset{0}{\overset{t}{\int\:}}{e}^{2}\left(t\right)dt$$

**Fig. 7:**
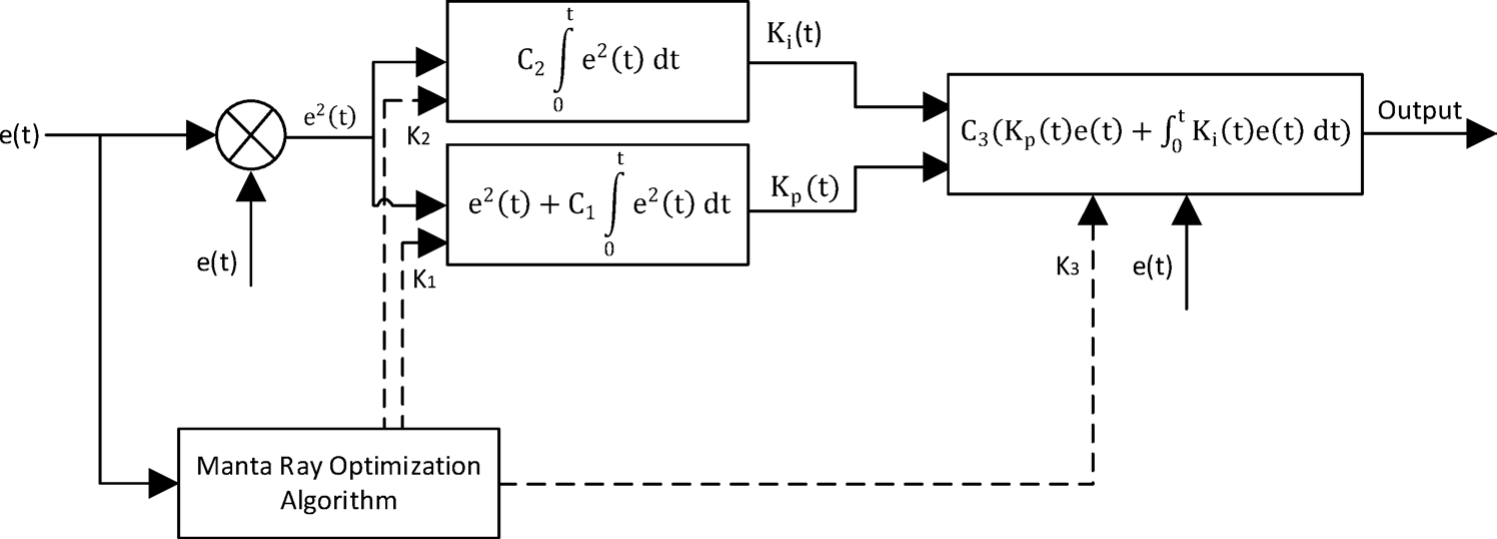
Adaptive PI Controller Block Diagram.

The Constants of API controller $\:{\text{C}}_{1},\:\:{\text{C}}_{2}\:and\:$ $\:{\text{C}}_{3}$ are optimized by MRFO Fig. 7 and the objective function for my controller is determined by (5).

**Fig. 8:**
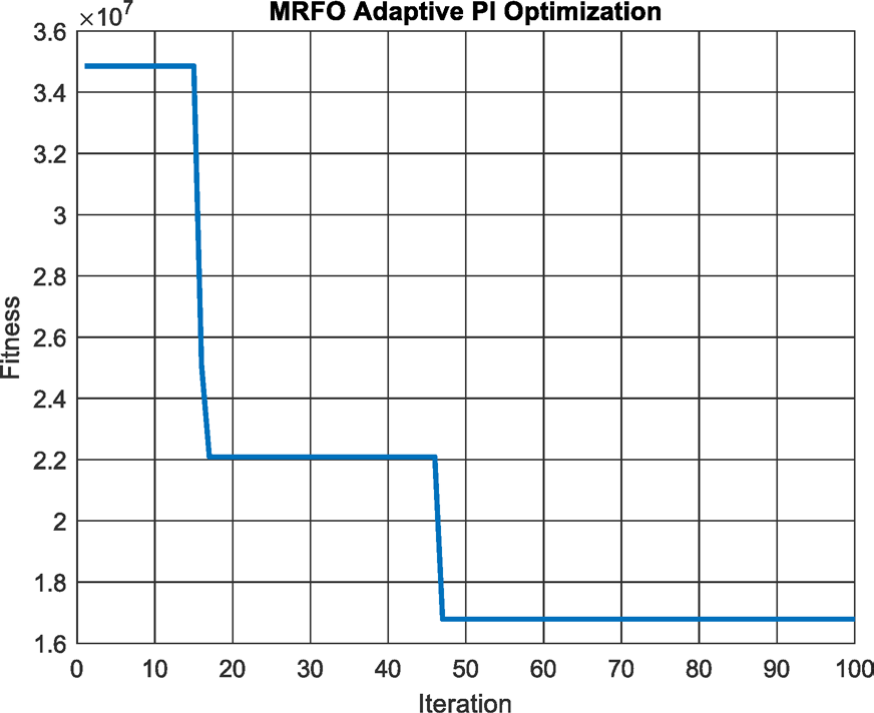
Performance vs. Iterations for API Controller.

MRFO algorithm is used with 20 population size in 100 iterations to minimize the integral square error of the PID controller of the MPPT Fig. 8 and the results were $\:{C}_{1}=\:$4.3290, $\:{C}_{2}=\:$0.6507 and $\:{C}_{3}=\:$0.0813.

### Single perceptron adaptive PI controller

 This controller is based on a single perceptron proportional-integral (SPPI) structure, and the output control signal^38^ is determined by

9 $$\:u\left(t\right)\:=\:m(k-1)\:+K\:\cdot\:\:\left[{g}_{1}\right(k)\:\cdot\:\:(e\left(k\right)-e(k-1))\:+{g}_{2}(k)\cdot\:e(k\left)\right]$$

where e(t) is the current tracking error, m(k-1) is the previous output, and K is a tuning gain that’s determine the system’s responsiveness. $\:{g}_{1}$(k) and $\:{g}_{2}$(k) represent adaptive proportional and integral gains. This formulation blends a PI control strategy with a simple learning mechanism inspired by single-layer perceptron behavior, aiming to improve MPPT performance by adapting control actions based on recent error dynamics. These equations capture the dynamic behavior of the power - voltage curve^39^. The error difference represents by (10). The current error represents by (11) The Change in output represents by (12). Slop estimation represents by (13)

10 $$\:{x}_{1}\left(k\right)=e\left(k\right)-\:e(k-1)$$

11 $$\:{x}_{2}\left(k\right)=\:e\left(k\right)$$

12 $$\:\varDelta\:m\left(k\right)=\:m\left(k\right)-m(k-1)$$

13 $$\:{\delta\:}_{e}\left(k\right)=\frac{{x}_{1}\left(k\right)}{\varDelta\:m\left(k\right)}$$

Update gains $\:{g}_{1}\left(k\right)$ and $\:{g}_{2}\left(k\right)$ represents by (14), (15), (16) and (17).

14 $$\:{y}_{1}\left(k\right)=\eta\:\:.\:e\left(k\right).{x}_{1}\left(k\right).{\delta\:}_{e}\left(k\right)$$

15 $$\:{y}_{2}\left(k\right)=\eta\:\:.\:e\left(k\right).{x}_{2}\left(k\right).{\delta\:}_{e}\left(k\right)$$

16 $$\:{g}_{1}\left(k\right)={g}_{1}(k-1)-\:{y}_{1}\left(k\right)$$

17 $$\:{g}_{2}\left(k\right)={g}_{2}(k-1)-\:{y}_{2}\left(k\right)$$

where $\:{\delta\:}_{e}\left(k\right)$ is error slope estimation, $\:{y}_{1}\left(k\right)$ and $\:{y}_{2}\left(k\right)$ are weight update contribution for the gains.

The gain of SP-API controller $\:K$ is optimized by manta ray algorism (MRFO) and the objective function for my controller is determined by (5).

**Fig. 9:**
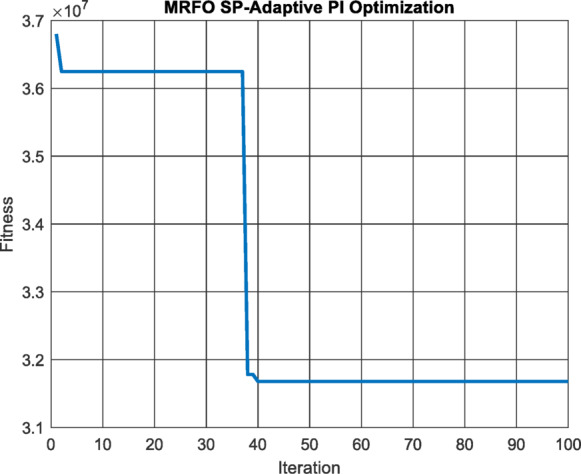
Performance vs. Iterations for SP-API Controller.

MRFO algorithm is used with 20 population size in 100 iterations to minimize the integral square error of the PID controller of the MPPT Fig. 9 and the results were $\:K=\:$457.5693.

### Set membership affine projection algorithm based PI controller

**Fig. 10:**
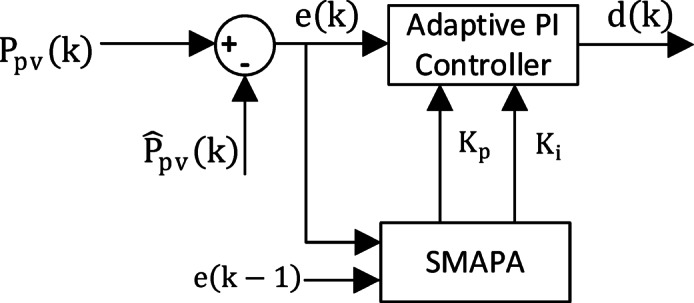
SMAPA Based Adaptive PI Controller Block Diagram.

The Set membership affine projection algorithm (SMAPA) is adaptive control technique^40,41^ used to online adjust Proportional and integral gains of the MPPT Controller to minimize fluctuation and ripples Fig. 10.The controller uses a vector input (18) where $\:e\left(t\right)\:$is the input tracking error^42^.

18 $$\:x\left(k\right)=\left[\:\:\varDelta\:e\right(k),\:\:e\left(k\right)+e(k-1)]$$

19 $$\:\varDelta\:e\left(k\right)=\:e\left(k\right)-e(k-1)$$

The predicted PV output power $\:{\widehat{P}}_{pv}\left(t\right)\:$is computed as (20).

20 $$\:{\widehat{P}}_{pv}\left(k\right)={w}^{T}\left(k\right).\:x\left(k\right)$$

21 $$\:w\left(k\right)=[{K}_{p}\left(k\right),\:{K}_{i}\left(k\right)]$$

22 $$\:e\left(k\right)=\:{P}_{pv}\left(k\right)-\:{\widehat{P}}_{pv}\left(k\right)$$

Where $\:{K}_{p}\left(k\right),\:{K}_{i}\left(k\right)$ are the adaptive PI gains which are then used to compute the control output (23).

23 $$\:u\left(k\right)={K}_{p}\left(k\right).\:\varDelta\:e\left(k\right)+{K}_{i}\left(k\right).(e\left(k\right)+e(k-1\left)\right)$$

## Case studies

In this part we study different case studies to compare the performance of PID controller, Adaptive PI controller, SP-API controller and SMAPA controller to evaluate the performance and robustness of the proposed MPPT controllers. The controllers’ parameters are summarized in Table 1; multiple case studies were conducted under varying environmental conditions. The PV system model was subjected to different levels of solar irradiance and ambient temperature to emulate realistic operating scenarios.

**Table 1:** Control parameters of the proposed MPPT Controllers.

Controller	Optimized parameters	Optimization/Update Method	Final Values
PID (MRFO)	$\:{K}_{p},{\:K}_{i},{\:K}_{d}$	MRFO (20 population, 100 iterations)	$\:{\text{K}}_{\text{p}}$= 2.1956, $\:{\text{K}}_{\text{i}}$= 0.7183, $\:{\:\text{K}}_{\text{d}}$​= 0.0012
Adaptive PI (API)	$\:{C}_{1},\:\:{C}_{2}\:,\:$ $\:{C}_{3}$, $\:{K}_{p},{\:K}_{i}$	MRFO-optimized constants + Online Update gains	$\:{\text{C}}_{1}$=4.3290, $\:{\text{C}}_{2}$​=0.6507, $\:{\text{C}}_{3}$​=0.0813, adaptive update for $\:{\text{K}}_{\text{p}},{\:\text{K}}_{\text{i}}$ with Eqs. (6–8)
SP-API	$\:K,\:\:{g}_{1}\:,\:$ $\:{g}_{2}$	MRFO tuning + perceptron update laws	K = 457.5693, learning weights updated using Eqs. (14–17)
SMAPA	$\:{K}_{p},{\:K}_{i}$	SMAPA-based online adaptation	Initialized to small positive values; updated using Eqs. (18–123)

### Temperature and irradiance variance

**Fig. 11:**
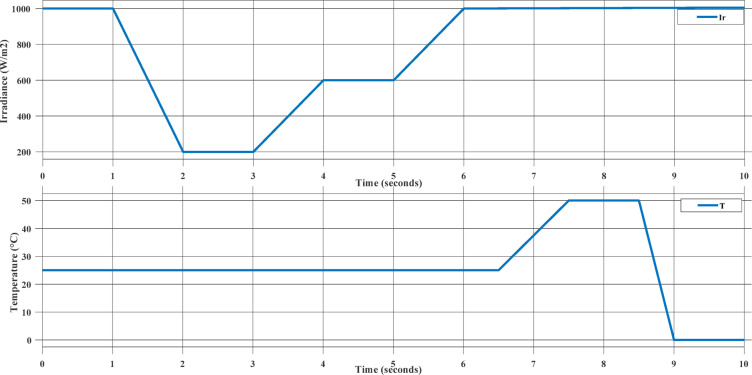
Irradiance and Temperature Input to the two PV Arrays.

This Case study represent the MPPT controllers’ performance under uniform environmental changes applied simultaneously to two identical PV arrays. Both irradiance and temperature vary with time, simulating realistic daily solar conditions such as passing clouds and rising daytime temperatures. Partial Shading with Varying Irradiance and Constant Temperature. The changes in temperature and irradiance are presented in Fig. 11.

**Fig. 12:**
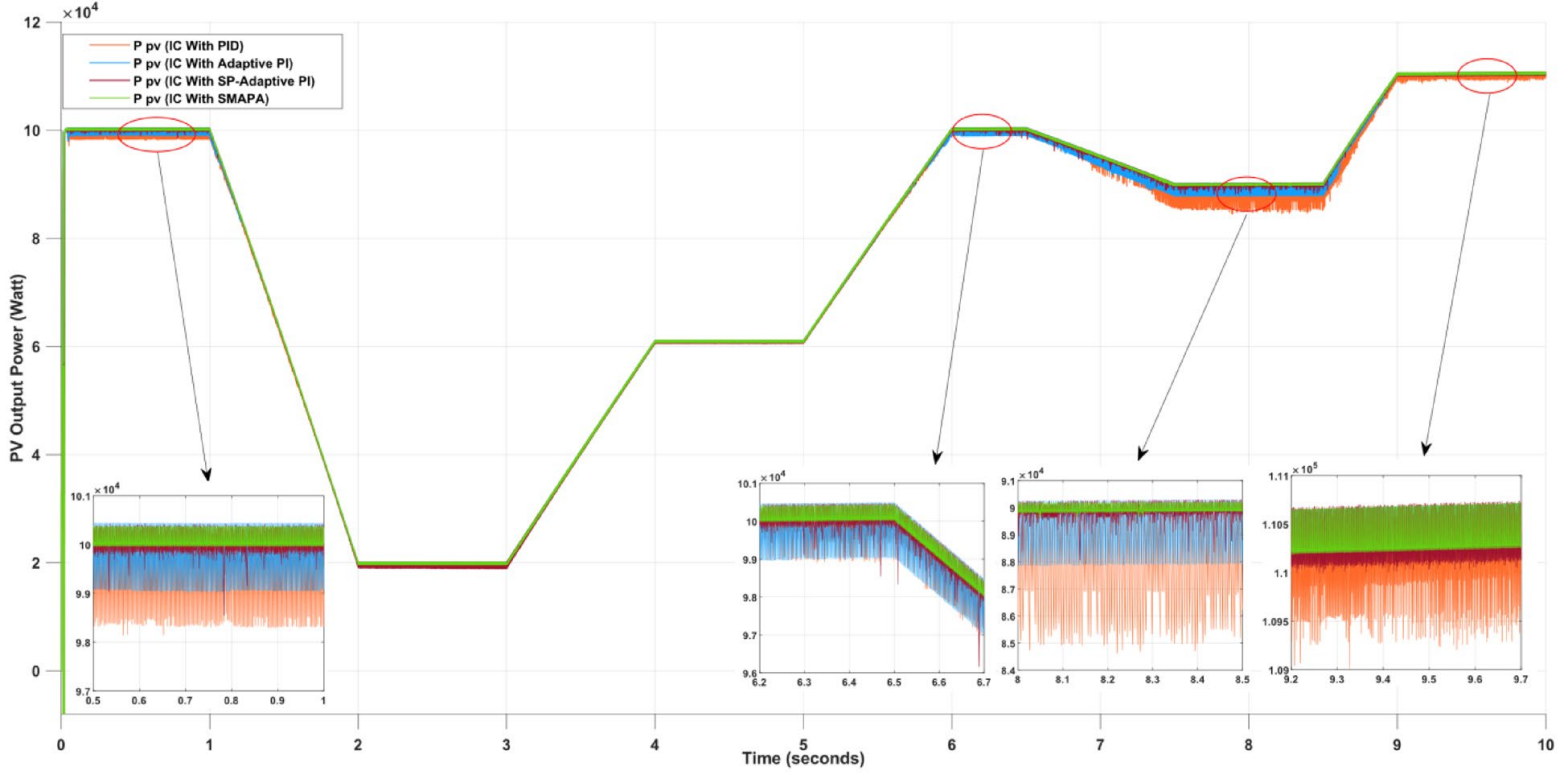
PV Output Power Using Different Controllers in MPPT.

Figure 12 and Table 2 illustrate the PV output power response of the four controllers under simultaneous temperature and irradiance variations. The PID controller shows noticeable oscillations and delayed settling time, which explains the higher ripple values (up to 5568 W) and efficiency drop below 97%. The Adaptive PI controller performs better, with smoother convergence and reduced oscillations, but still exhibits moderate ripple levels and efficiency limited to 97–98%. In contrast, the SP-API controller demonstrates rapid convergence to the MPP, maintaining high power output above 110 kW with reduced ripples (~ 509–1028 W). The SMAPA controller outperforms all others by delivering the most stable waveform, with nearly ripple-free operation (~ 223 W), minimal losses (~ 0.2%), and near-ideal efficiency (~ 99.8%), confirming its superior adaptability.

**Table 2:** Difference controllers performance during temperature and irradiance Variance.

MPPT techniques	Interval (0 to 1 s)	Interval (7.5 to 8.5 s)	Interval (9 to 10 s)
Average steady state power (W)			
PID Controller	99,240	87,399	109,801
Adaptive PI Controller	99,740	89,029	110,029
SP-API Controller	100,076	89,669	110,480
SMAPA Controller	100,612	90,019	110,490
Ripples (W)			
PID Controller	2181	5568	1492
Adaptive PI Controller	1398	2308	445
SP-API Controller	509	1028	658
SMAPA Controller	377.6	327.4	223
Energy losses (%)			
PID Controller	1.75	3.11	2.37
Adaptive PI Controller	1.69	2.46	1.30
SP-API Controller	0.92	0.59	0.48
SMAPA Controller	0.19	0.21	0.20
Efficiency (%)			
PID Controller	98.25	96.89	97.63
Adaptive PI Controller	98.31	97.54	98.70
SP-API Controller	99.08	99.41	99.52
SMAPA Controller	99.81	99.79	99.80

### Partial shading

**Fig. 13:**
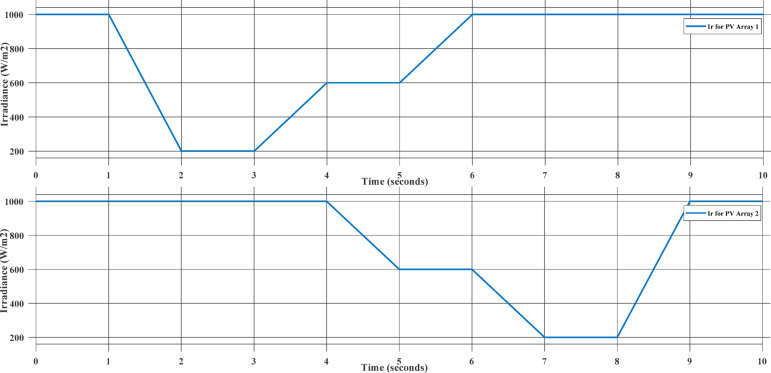
Irradiance Input to PV Array 1 and 2 During Partial Shading.

**Fig. 14:**
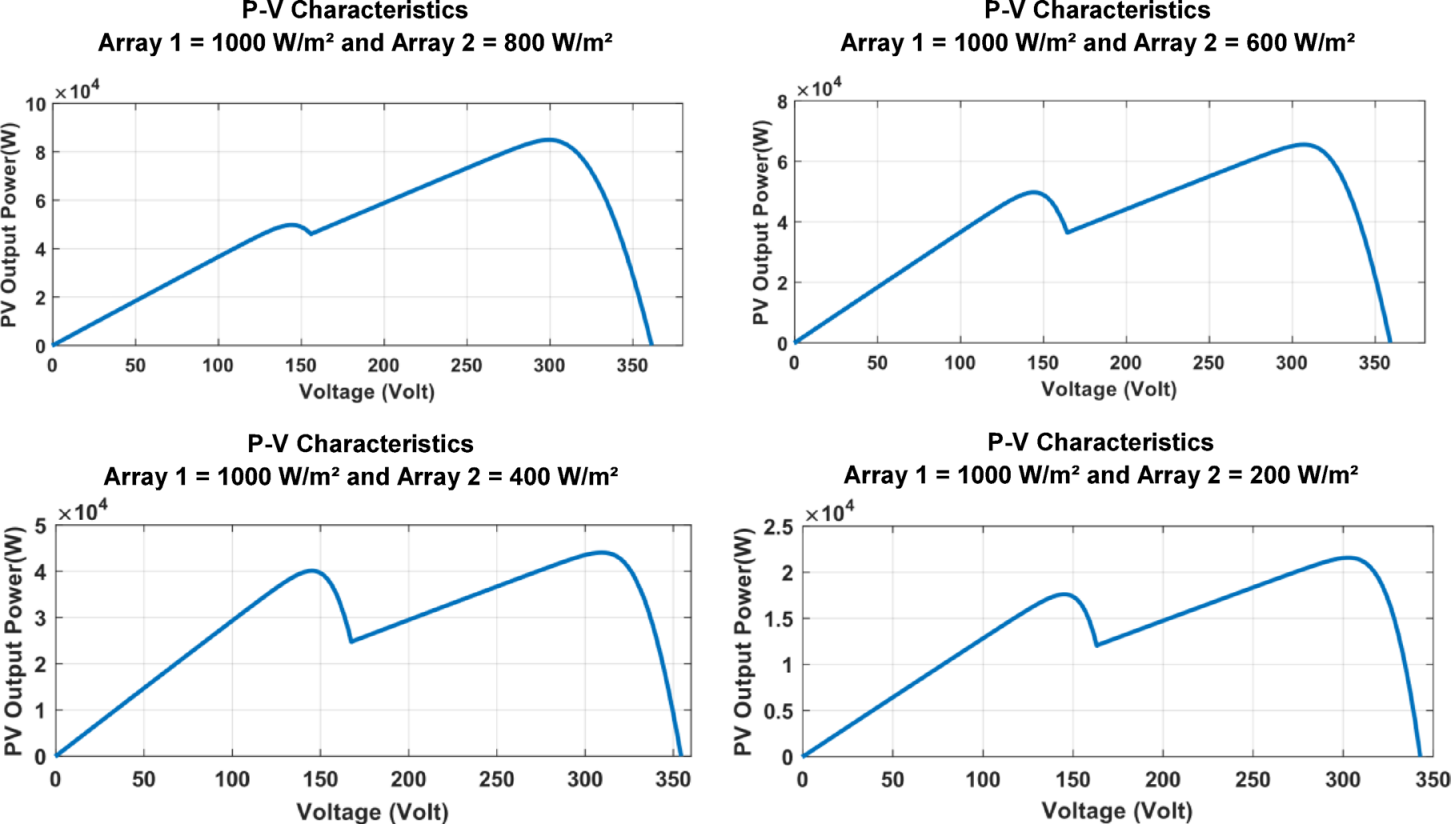
P-V Curves for The Two Arrays Under Different Irradiances.

In this case study, a partial shading condition is simulated to evaluate the performance of the MPPT controllers when two PV arrays operate under constant temperature but variable irradiance. Both PV Array 1 and PV Array 2 are maintained at a fixed temperature of 25 °C, while their irradiance levels differ to replicate real-world partial shading scenarios. Such variation is typically caused by shadowing from nearby structures or transient effects like passing clouds. The irradiance changes applied to PV Array 1 and PV Array 2 are illustrated in Fig. 13. P-V curves for the two arrays under different irradiances are shown on Fig. 14.

**Fig. 15:**
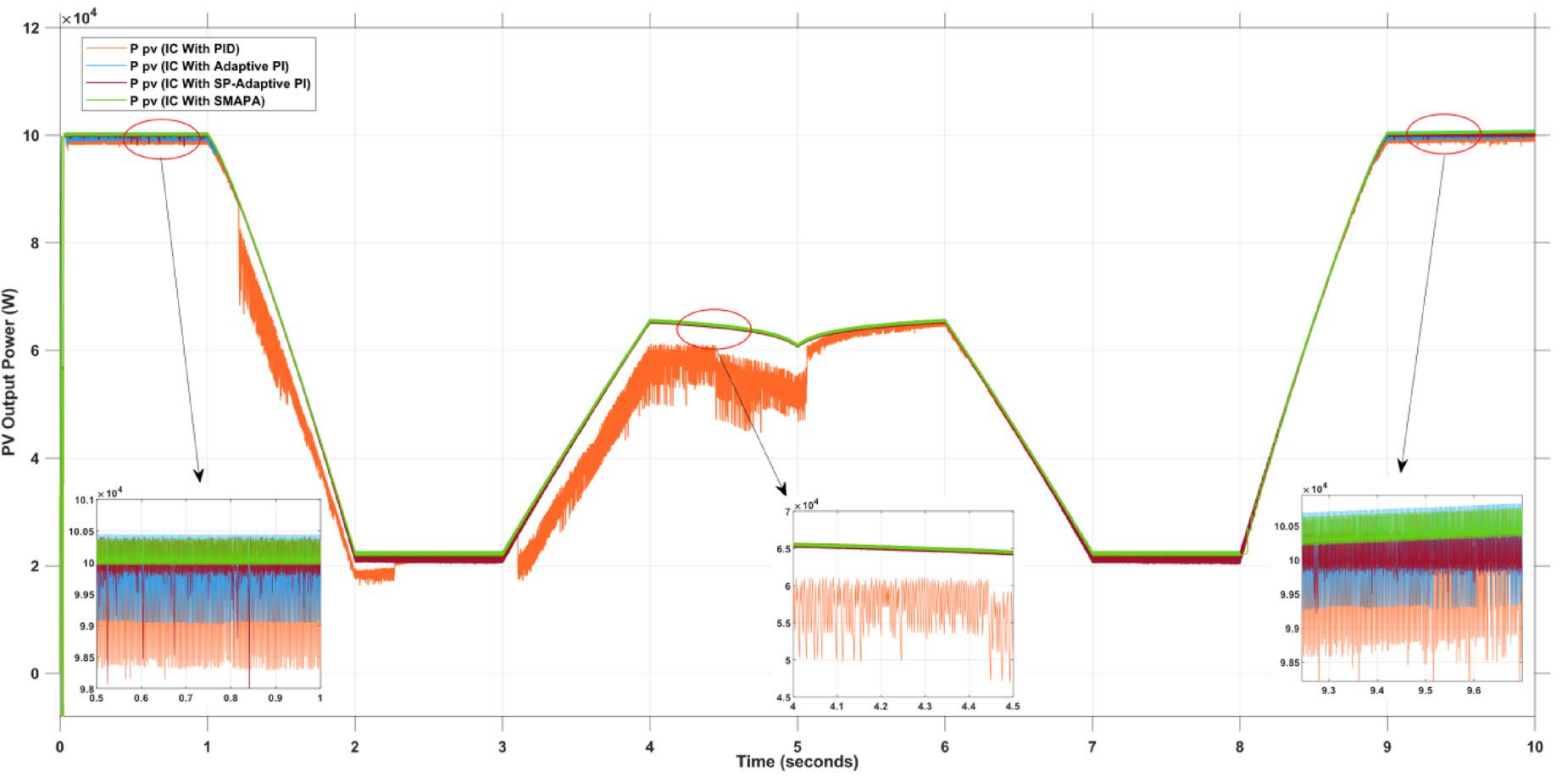
PV Output Power Using Different Controllers in MPPT During Partial Shading.

Figure 15; Table 3 present the PV power under partial shading conditions. The PID controller shows sharp power drops and instability during irradiance mismatch, reflecting its inability to track the global MPP. The Adaptive PI controller manages smoother recovery but still loses efficiency due to limited adaptability. The SP-API controller performs robustly, with higher stability and efficiency above 99%, though it still exhibits moderate ripples under sudden shading changes. The SMAPA controller again provides the most effective response, as evident from its smooth power waveform, negligible ripples, and consistent tracking of the global MPP, maintaining nearly constant efficiency close to 99.8%.

**Table 3:** Difference controllers performance during partial Shading.

MPPT techniques	Interval (0 to 1 s)	Interval (4 to 5 s)	Interval (9 to 10 s)
Average steady state power (W)			
PID Controller	99,240	55,140	99,323
Adaptive PI Controller	99,740	64,430	99,953
SP-API Controller	100,076	64,491	100,092
SMAPA Controller	100,612	65,660	100,582
Ripples (W)			
PID Controller	2181	10,627	2458
Adaptive PI Controller	1398	890	1444
SP-API Controller	509	519	876
SMAPA Controller	377.6	321	386
Energy losses (%)			
PID Controller	1.75	16.46	1.67
Adaptive PI Controller	1.69	2.48	1.44
SP-API Controller	0.92	2.29	0.9
SMAPA Controller	0.19	0.24	0.14
Efficiency (%)			
PID Controller	98.25	83.54	98.33
Adaptive PI Controller	98.31	97.52	98.56
SP-API Controller	99.08	97.71	99.10
SMAPA Controller	99.81	99.76	99.86

**Fig. 16:**
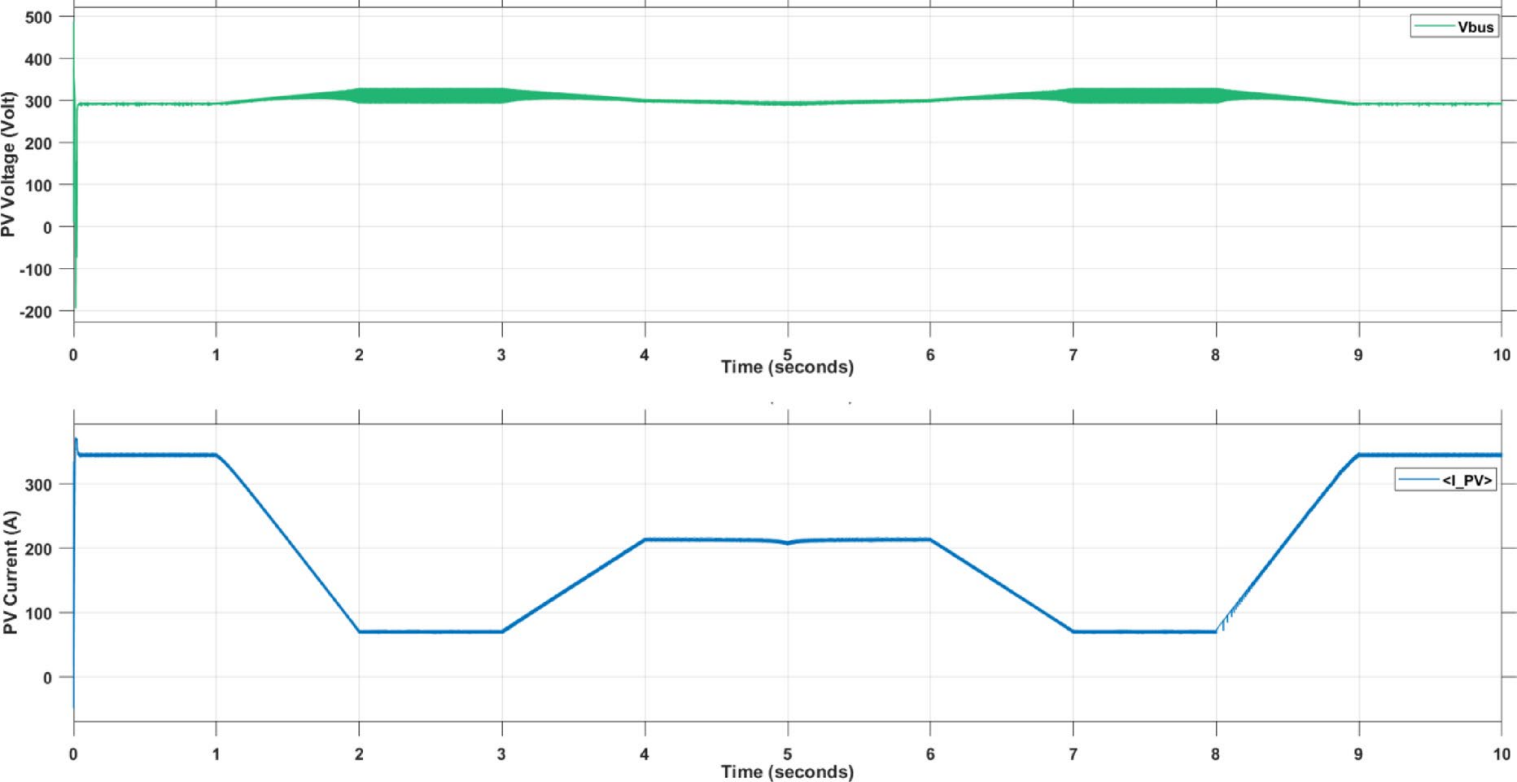
PV Output Voltage and Current During Using SMAPA.

Figure 16 shows the PV output voltage and current for the most superior technique, SMAPA.

**Fig. 17:**
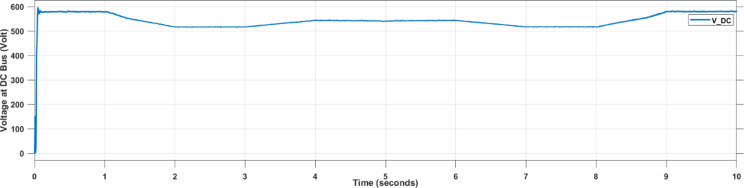
Voltage at DC Bus During Using SMAPA.

Figure 17 presents the DC bus voltage response after boost converter when using the SMAPA technique. It demonstrates that SMAPA maintains stable and well-regulated DC voltage with minimal fluctuation.

**Fig. 18:**
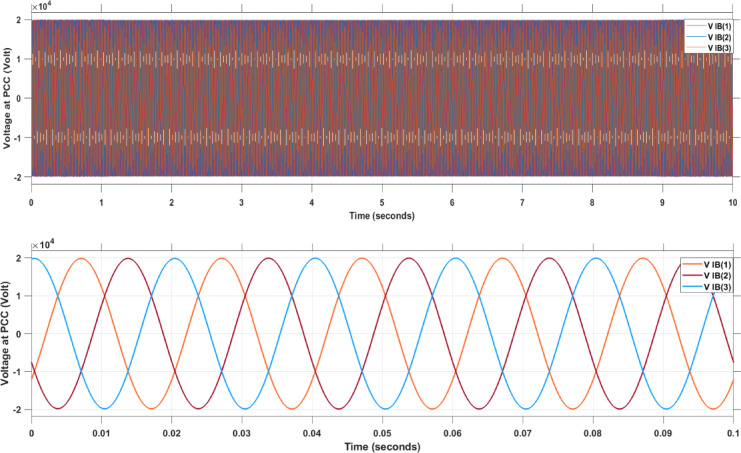
AC Voltage at PCC.

Figure 18 shows the voltage at the point of common coupling (PCC) when applying the SMAPA technique, presented for two different time periods: 10 s and 0.1 s.

The effectiveness of the proposed SMAPA-based MPPT technique is evaluated through a comparative analysis with several state-of-the-art strategies, as summarized in Table 4. Key evaluation criteria include the control strategy, tracking speed, tracking accuracy, system complexity, implementation cost, efficiency, grid integration capability and Real-Time Adaptation. These factors collectively determine the robustness, adaptability, and practical feasibility of each MPPT technique under varying operating conditions.

**Table 4:** Comparison of the various MPPT techniques.

Technique	Tracking Accuracy	Fluctuation around MPP	Tracking Speed	Complexity	Grid Integration	Cost	Efficiency	Real-Time Adaptation
P&O^3–5^	Medium	High	Medium	Simple	✗ No	Low	~ 96%	✗ No
INC^3–5^	Medium	Medium	Medium	Simple	✓ Yes	Low	~ 97%	✗ No
Fuzzy-PID^7^	High	Low	High	Complex	✓ Yes	Medium	98.20%	Semi
Neuro-Fuzzy IC^8^	High	Low	High	Complex	✓ Yes	High	98.60%	✗ No
SVM-Based^9^	High	Medium	Medium	Complex	✓ Yes	High	98.40%	✗ No
Cuttlefish Algorithm^10^	High	Low	High	Complex	✓ Yes	High	98.80%	✗ No
ANN-Based^12^	High	Medium	High	Complex	✓ Yes	High	98%	✗ No
LSTM–ANN–FLC Hybrid^13^	High	Low	High	Complex	✓ Yes	High	99%	✗ No
MRAC–Fuzzy^14^	High	Low	High	Medium	✓ Yes	Medium	98.70%	✓ Yes
Hybrid CT-P&O + MPC^15^	High	Low	High	Complex	✓ Yes	Medium	98.90%	Partial
TSA–PSO Hybrid^19^	High	Low	High	Complex	✓ Yes	High	99.10%	✗ No
Lyapunov MRAC / Robust MRAC^23,24^	High	Very Low	High	Complex	✓ Yes	Medium	99.6%	✓ Yes
EMRAC^27^	High	Very Low	High	Medium	✓ Yes	Medium	98.30%	✓ Yes
RMRAC (Recent)^28^	High	Very Low	Very High	Medium	✓ Yes	Medium	99.75%	✓ Yes
*MRFO-PID* *(This Study)*	Medium	Low	Medium	Simple	✓ Yes	Low	98.25%	✓ Yes
*Adaptive PI* *(This Study)*	Medium	Low	Medium	Simple	✓ Yes	Low	98.31%	✓ Yes
*SP-API* *(This Study)*	High	Very Low	Very High	Medium	✓ Yes	Medium	99.08%	✓ Yes
Proposed SMAPA–IC *(This Study)*	High	Very Low	Very High	Complex	✓ Yes	Medium	99.80%	✓ Yes

## Conclusion

This Study compared four adaptive controllers—PID (MRFO-tuned), Adaptive PI, SP-API, and SMAPA—integrated with the incremental conductance (IC) method for MPPT in a grid-connected PV system under both uniform and partial shading conditions. The results demonstrate that while the PID and Adaptive PI controllers can provide acceptable performance, they suffer from significant oscillations, higher power losses, and reduced efficiency under rapidly changing irradiance and temperature. The SP-API controller showed improved adaptability with efficiency consistently above 99%, reduced ripples, and stable dynamic response. However, the SMAPA-based PI controller achieved the most robust performance, maintaining near-ideal efficiency (~ 99.8%), negligible ripples (~ 223 W), and minimal energy loss (~ 0.2%) even under partial shading scenarios.

The key insight from this work is that online adaptive learning mechanisms (SP-API and SMAPA) outperform both conventional and metaheuristic-tuned controllers, particularly when the PV system is subject to uncertainties and dynamic variations. This indicates that future MPPT designs for grid-connected PV should prioritize adaptive frameworks that can continuously adapt to environmental changes, thereby ensuring maximum energy harvesting, stability, and reliability.

A limitation of this study is that the proposed SMAPA-based MPPT controller was validated only through simulation without Hardware-in-the-Loop (HIL) implementation. In addition, the controller exhibits a small steady-state error that slightly affects its convergence to the exact maximum power point, and its mathematical formulation is relatively complex, which may increase computational load in real-time applications. Future work will therefore focus on implementing the controller to verify its real-time performance under practical grid-connected and partial shading conditions.

## Data Availability

The datasets used and/or analyzed during the current study are available from the corresponding author on reasonable request.
